# Baseline splenic volume as a biomarker for clinical outcome and circulating lymphocyte count in gastric cancer

**DOI:** 10.3389/fonc.2022.1065716

**Published:** 2023-01-30

**Authors:** Ziyang Zeng, Zhen Liu, Jie Li, Juan Sun, Mingwei Ma, Xin Ye, Jianchun Yu, Weiming Kang

**Affiliations:** Department of General Surgery, Peking Union Medical College Hospital, Chinese Academy of Medical Sciences and Peking Union Medical College, Beijing, China

**Keywords:** gastric cancer, spleen, prognosis, immune system, lymphocyte

## Abstract

**Background:**

The spleen is the largest peripheral lymphoid organ in the body. Studies have implicated the spleen in the development of cancer. However, it is unknown whether splenic volume (SV) is associated with the clinical outcome of gastric cancer.

**Methods:**

Data of gastric cancer patients treated with surgical resection were retrospectively analyzed. Patients were divided into three groups: underweight, normal-weight and overweight. Overall survival was compared in patients with high and low splenic volume. The correlation between splenic volume and peripheral immune cells were analyzed.

**Results:**

Of 541 patients, 71.2% were male and the median age was 60. Underweight, normal-weight and overweight patients accounted for 5.4%, 62.3% and 32.3%, respectively. High splenic volume was associated with unfavorable prognosis across the three groups. In addition, the increase of splenic volume during neoadjuvant chemotherapy was not associated with prognosis. The baseline splenic volume was negatively correlated with lymphocytes (r=-0.21, p<0.001) and positively correlated with NLR (neutrophil-to-lymphocyte ratio) (r=0.24, p<0.001). In a group of patients (n=56), splenic volume was found to have negative correlation with CD4+T cells (r=-0.27, p=0.041) and NK cells (r=-0.30, p=0.025).

**Conclusions:**

The presence of high splenic volume is a biomarker of unfavorable prognosis and reduced circulating lymphocytes in gastric cancer.

## Introduction

Gastric cancer is the fifth most common and the fourth most lethal cancer in the world (1). Despite the development in conventional therapeutic strategies, including surgery, radiotherapy and chemotherapy, the overall prognosis remained poor, with an estimated 5-year survival rate of 45% in advanced stage (2). The past decade has witnessed the emergence of immunotherapy in cancer therapy, and immune checkpoint inhibitors (ICIs) have emerged as a new therapeutic strategy in gastric cancer (3). The ATTRACTION-2 study has demonstrated prolonged survival by anti-PD1 therapy in the third-line setting of gastric cancer (4), but the clinical benefit was observed only in a minority of patients. When applied as monotherapy, different trials of anti-PDL1 showed a range of response rates from 11% to 22% in the unselected patients (4–6). However, in patients with MSI-H, which is characterized by high number of tumor neoantigens, the objective response rates ranged from 47% to 57% (6–8). EBV-positive GC is another particular subtype associated with enhanced clinical benefit, with a rather wide range of objective response rates of 25% to 100% (9–13) in various settings. The highly inflamed microenvironment of MSI-H and EBV-positive tumors can partly explain the favorable response of ICIs (14). Accordingly, high tumor mutational burden (TMB-H) which facilitates immune recognition is also predictive of improved clinical response and overall survival (15). However, these predictive biomarkers currently in use are largely derived from immune microenvironment within the tumor, and does not fully account for the systemic immune landscape.

Recent studies suggested that successful immunotherapy depends on the system-wide immune response, rather than the local response within tumor (16). Antitumor immune response cannot proceed without communication with the periphery (17). Studies have found that PD1 and PDL1 blockade drove new T cell clones into the tumor microenvironment, instead of reinvigorating pre-existing effector T cells (18, 19). And the crossing of survival curves between anti-PDL1 and chemotherapy in the phase 3 KEYNOTE-061 and KEYNOTE-062 trials also suggested a period needed for awakening the immune system to attack tumor cells after the initiation of ICIs (7, 8), addressing the importance of immunological background in driving and sustaining efficacious immunotherapy responses.

Also, it is now accepted that conventional chemotherapy can augment antitumor immune response by increasing antigenicity and adjuvanticity of cancer cells, and rebound replenishment of immune cell pools following lymphodepletion (20, 21). In the setting of chemotherapy, therapeutic efficacy was found to substantially correlate with lymphocyte and NK cell counts in the periphery (22, 23). Studies showed that some chemotherapeutic drugs appeared more effective in immuno-competent hosts than in immuno-deficient counterparts (20). These data suggested the fundamental role of global immune macroenvironment (17, 24, 25) as the basis for various therapies. Immunity is coordinated across diverse cell types and tissues. And with the advent of immunotherapy, either used alone or in combination with chemotherapy, a thorough understanding of the systemic immunity is needed to better harness the potentiality of immunotherapy to treat gastric cancer.

As the largest peripheral lymphoid organ in the body, the spleen is a pivotal site for innate and adaptive immune response, and is estimated to host one third of the immune cells in the body (26). The spleen is now receiving more attention in the context of cancer for its capacity in generating tumor-associated macrophages (TAMs) and tumor-associated neutrophils (TANs) (27, 28). In a few solid tumors (29, 30), splenic volume proved to be a prognostic biomarker that was associated with the immune status of patients.

In this retrospective study, we focused on the spleen and aimed to determine the prognostic role of baseline splenic volume in patients with gastric cancer. Further, we explored whether the splenic volume was correlated to peripheral immune cells.

## Methods

### Patients

Data of patients who underwent surgical resection for gastric cancer between January 2015 and March 2018 in Peking Union Medical College Hospital (PUMCH) were retrospectively reviewed. Clinical data were extracted from an electronic database. The baseline CT scan images were retrieved from a workstation (Syngo MMWP; Siemens Healthcare). Besides the baseline CT, we also retrieved CT scans after neoadjuvant chemotherapy was completed (evaluated before surgery) in available cases, to compare the change of splenic volume (△SV) during neoadjuvant chemotherapy and evaluated the prognostic effects.

As the spleen serves a reservoir of immune cells, we also compared the difference of circulating immune cell populations in patients with high SV and low SV. In a subset of patients in our cohort, immunophenotyping of blood lymphocytes was analyzed by flow cytometry (NAVIOS, Bechman Coulter, USA). Freshly collected EDTA-anticoagulated whole blood was incubated and tested with a panel of monoclonal antibodies directed against CD3, CD4, CD8, CD19, CD16 and CD56. Lymphocyte subsets were calculated using a dual-platform method with the white blood cell counts and lymphocyte differentials obtained from blood routine tests of the same specimen. All blood tests were performed at baseline, which was before the initiation of treatments.

The WHO criteria for obesity was adopted to classify the total patients into 3 groups: underweight (<18.50kg/m²), normal weight (18.5-24.99 kg/m²) and overweight (≥25.00kg/m²) (31). This is because the volume of spleen is proportional to the patients’ BMI. To allow comparisons of high and low splenic volume only made within patients with similar BMI, we conducted the comparative study in each BMI group, respectively.

Before surgery, neoadjuvant chemotherapy (NAC) was administered depending on clinical assessments at baseline evaluation. The indication for NAC in our center was locally advanced gastric cancer with clinical T stage ≥ 3 or N stage ≥ 1. Radical total or subtotal gastrectomy with D2 lymphadenectomy were performed for curative resection. Palliative resection was performed in the presence of major symptoms for patients with non-curative gastric cancer. The tumor size was defined as the longest diameter of the tumor mass visible on pathological examination. Pathologic staging was assigned based on the 8th edition of the American Joint Committee on Cancer (AJCC) staging manual. After surgery, patients were followed up every 3-6 months for two years and every one year thereafter. The study was approved by the institutional review boards of PUMCH (K1447). Written informed consent was waived because of the retrospective nature of this study.

### Definition of postoperative complications

All 30-day postoperative adverse events were graded by the Clavien-Dindo system (32, 33). Adverse events classified as Clavien-Dindo grade II or higher were defined as postoperative complications. Postoperative complications included abdominal complications: anastomotic leakage, intra-abdominal infection, peritoneal effusion, bleeding, pancreatic fistula, chylous leakage, delayed gastric emptying, mechanical bowel obstruction, paralytic ileus and delayed wound dehiscence; respiratory complications: pneumonia, pleural effusion and pulmonary embolism; and infection with unknown causes, cardiovascular complications and urinary complications.

### Spleen volumetry

The baseline CT images were analyzed on the workstation and the splenic volume (SV) of the patients was measured by an experienced analyzer. Briefly, the margin of the spleen was manually contoured in each CT image to calculate the area that was enclosed, taking into account the slice thickness. Then, the volume of each slice of the spleen from the upper pole to the lower pole was summed to obtain the total volume of the spleen (**Supplementary Figure 1**). To ensure reproducibility, the same analyzer repeated the measurements on one hundred randomly selected patients to calculate intra-observer variability. Two analyzers independently performed measurements on twenty randomly selected patients to calculate inter-observer variability. The agreement between analyses were calculated using concordance correlation coefficients (CCC).

### Statistical analysis

All statistical analyses were performed using the R software (4.1.3). Concordance correlation coefficients (CCC) were calculated using the R package ‘cccrm’. The comparisons between continuous data were performed using Mann Whitney test or Kruskal-Wallis test. Paired samples Wilcoxon test was used for the comparison of splenic volume before and after neoadjuvant chemotherapy. We used the maximally selected log-rank statistics to choose the optimal cutoff for splenic volume with the R package ‘maxstat’ (34). Patients were divided into a high SV and low SV group by a candidate splenic volume threshold. The cutoff that best separated patient outcome with the maximum log-rank statistics and minimum P value was selected as the optimal cutoff. Overall survival was defined as the time from surgery to death. Kaplan-Meier curves with log-rank tests were used to compare survival between different groups. Cox proportional hazard regression model was performed for multivariate analyses. In multivariate Cox analysis, clinically important variables were entered into the model. The correlations between the splenic volume and celluar blood components were evaluated with Spearman’s rank correlation coefficient. A two-sided P value less than 0.05 was considered to indicate statistical significance.

## Results

### Patient characteristics

**Figure 1:**
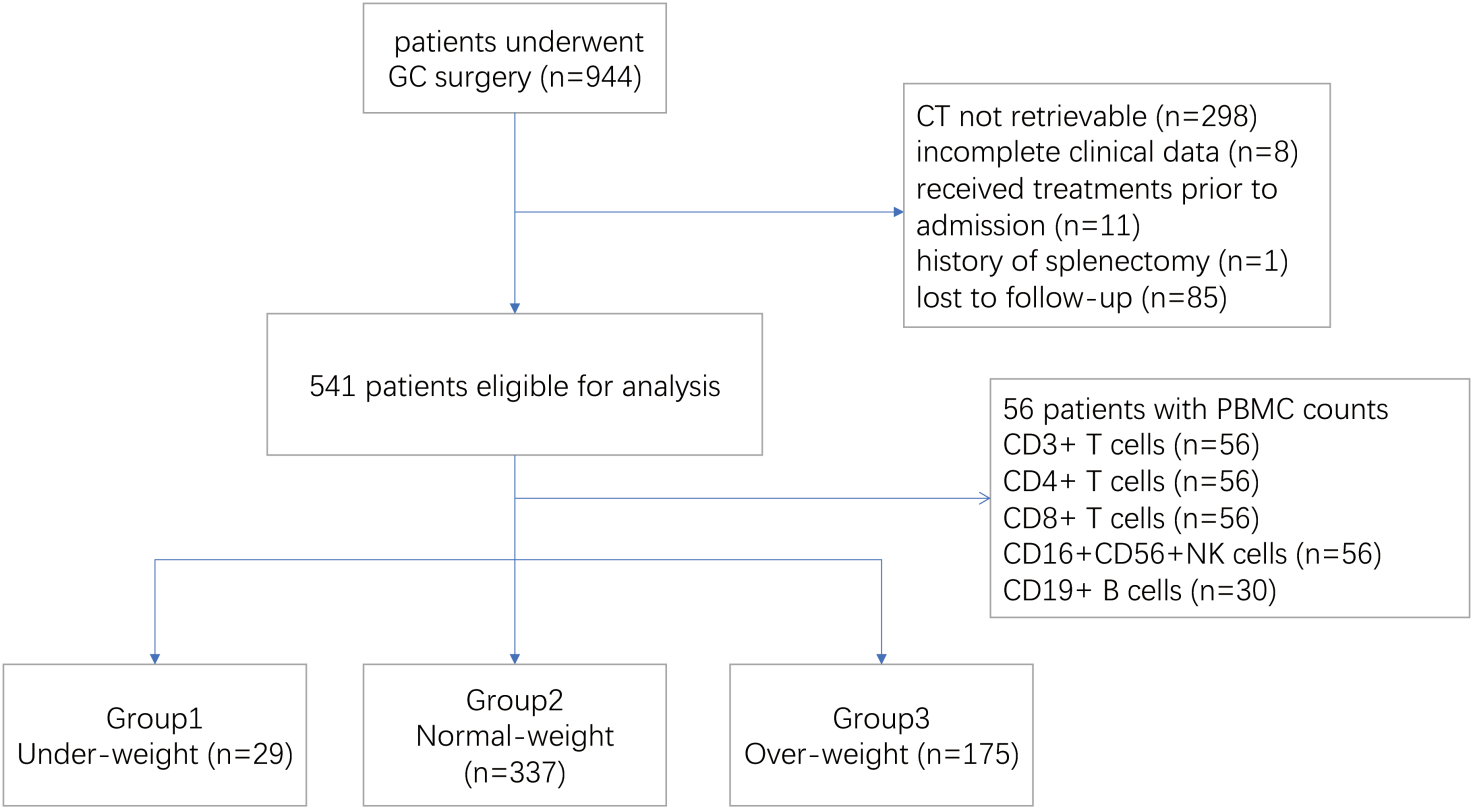
Study flowchart.

Between January 2015 and March 2018, a total of 944 patients who had surgical resection for gastric cancer were screened, of whom 541 were eligible for the study. Patients were excluded because of incomplete clinical data (n=8), unavailable CT scan (n=298), previous splenectomy (n=1), prior treatment before admission (n=11) and follow-up for less than two years (n=85) (**Figure 1**). In this study, 56 patients were tested for lymphocyte subsets before treatment initiation, including CD3/CD4/CD8+T cells and CD16+CD56+NK cells, and 30 patients were tested for peripheral CD19+B cells. And the association between splenic volume and immunocyte subsets were investigated in this group of patients (**Figure 1**).

Of 541 patients enrolled, the median (IQR) age was 60 (53–67), and most patients were male (385 [71.2%]). The median (IQR) BMI was 23.4 (21.3-25.6) kg/m². In the total population, 157 (29.0%) had received neoadjuvant chemotherapy before surgery. The proportion of total and partial resection was 64.3% and 35.7%, respectively. Adjuvant chemotherapy was administered in 314 (58.0%) patients post surgery. The median (IQR) value of the CT-based splenic volume was 167.0 (124.9 to 224.6) ml, with a substantial agreement for inter-reader variability (CCC [concordance correlation coefficient]:0.997, 95%CI = 0.994–0.999) and intra-reader variability (CCC:0.997, 95%CI = 0.996–0.998). Baseline patient characteristics are detailed in **Table 1**.

**Table 1:** Patient characteristics.

Characteristics	Total	Underweight	Normal-weight	Overweight
Age, median (IQR), y	60.0 (53-67)	59.0 (54-70)	60.0 (52-67)	61.0 (54-67)
Male	385 (71.2%)	17 (58.6%)	225 (66.8%)	143 (81.7%)
BMI, median (IQR), kg/m²	23.4 (21.3-25.6)	17.4 (16.6-18.0)	22.2 (20.9-23.7)	26.4 (25.7-28.1)
Tobacco				
Never	291 (53.8%)	15 (51.7%)	188 (55.8%)	88 (50.3%)
Former	115 (21.3%)	5 (17.2%)	71 (21.1%)	39 (22.3%)
Current	135 (25.0%)	9 (31.0%)	78 (23.1%)	48 (27.4%)
Neoadjuvant chemotherapy	157 (29.0%)	8 (27.6%)	104 (30.9%)	45 (25.7%)
Tumor location				
Lower third	290 (53.6%)	17 (58.6%)	193 (57.3%)	80 (45.7%)
Middle third	147 (27.2%)	4 (13.8%)	95 (28.2%)	48 (27.4%)
Upper third	104 (19.2%)	8 (27.6%)	49 (14.5%)	47 (26.9%)
Tumor size, median (IQR), cm	3.0 (2.0-4.0)	3.5 (2.5-5.2)	3.0 (2.0-4.5)	2.5 (2.0-4.0)
Tumor grade				
Poorly differentiated	377 (69.7)	21 (72.4)	244 (72.4)	112 (64.0)
Well-moderately differentiated	164 (30.3)	8 (27.6)	93 (27.6)	63 (36.0)
T stage				
T_1_	186 (34.4)	8 (27.6)	106 (31.5)	72 (41.1)
T_2_	86 (15.9)	4 (13.8)	54 (16.0)	28 (16.0)
T_3_	170 (31.4)	11 (37.9)	105 (31.2)	54 (30.9)
T_4_	99 (18.3)	6 (20.7)	72 (21.4)	21 (12.0)
Lymph node metastasis				
No	286 (52.9)	12 (41.4)	164 (48.7)	110 (62.9)
Yes	255 (47.1)	17 (58.6)	173 (51.3)	65 (37.1)
Distant metastasis				
No	530 (98.0)	28 (96.6)	329 (97.6)	173 (98.9)
Yes	11 (2.0)	1 (3.4)	8 (2.4)	2 (1.1)
TNM Stage				
I	220 (40.7%)	8 (27.6%)	124 (36.8%)	88 (50.3%)
II	146 (27.0%)	9 (31.0%)	97 (28.8%)	40 (22.9%)
III	164 (30.3%)	11 (37.9%)	108 (32.0%)	45 (25.7%)
IV	11 (2.0%)	1 (3.4%)	8 (2.4%)	2 (1.1%)
Postoperative complications				
No	419 (77.4)	25 (86.2)	270 (80.1)	124 (70.9)
Yes	122 (22.6)	4 (13.8)	67 (19.9)	51 (29.1)
Adjuvant chemotherapy	314 (58.0)	13 (44.8)	208 (61.7)	93 (53.1)
Baseline splenic volume, median (IQR), ml	167.0 (124.9-224.6)	121.3 (86.9-153.5)	159.7 (119.1-203.6)	206.5 (154.1-252.4)

**Figure 2:**
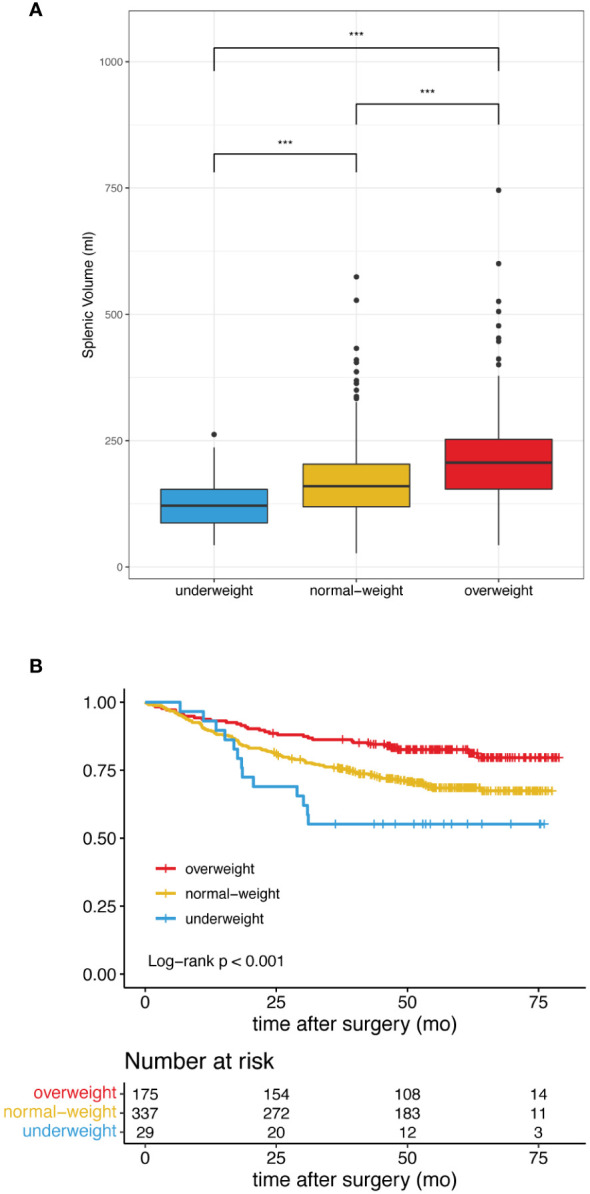
Difference of splenic volume and overall survival in patients classified as underweight, normal-weight and overweight. **(A)** High BMI is associated with Increased splenic volume. *** indicates p< 0.001. **(B)** Patients with high BMI showed improved overall survival.

Based on the BMI stratification, there were 32.3%, 62.3% and 5.4% patients in the overweight, normal-weight and underweight group, respectively. Underweight, normal weight and overweight patients not only showed different splenic volume (median 121.3 ml vs 159.7 ml vs 206.5 ml, p<0.001) (**Figure 2A**), but also different long-term survival patterns (**Figure 2B**). Consistent with a large cohort study of the prognostic effect of BMI in gastric cancer (35), the survival curves in our study showed that the overweight patients were associated with more favorable clinical outcome, followed by normal-weight and underweight patients (**Figure 2B**). To analyze the prognostic effects of different splenic volume in patients of comparable body size, we divided the study population into underweight (<18.50kg/m²), normal-weight (18.5-24.99 kg/m²) and overweight (≥25.00kg/m²) to study the survival differences between high SV and low SV in each BMI subgroup independently (**Table 1**).

### Survival analysis

**Figure 3:**
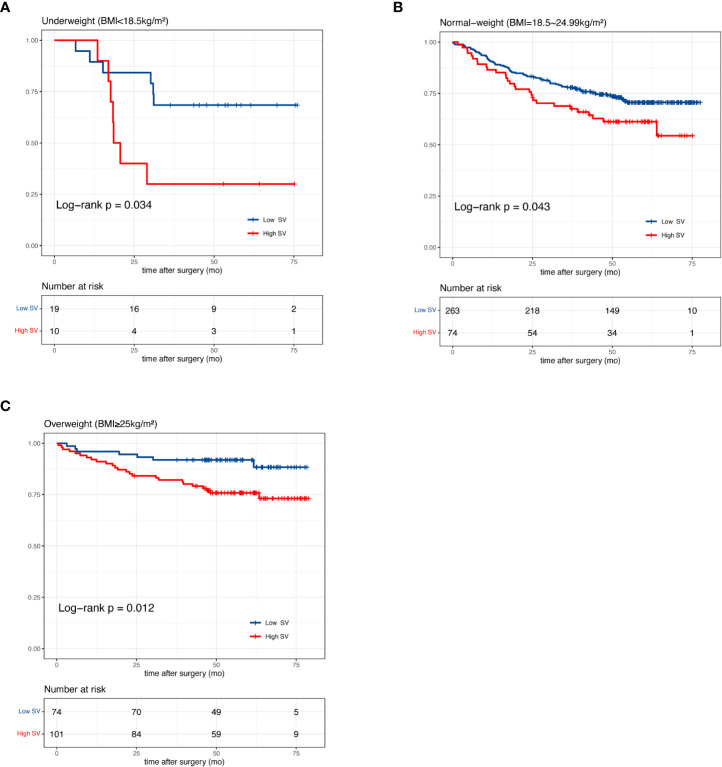
Kaplan-Meier curves of patients with high and low splenic volume in **(A)** underweight **(B)** normal-weight and **(C)** overweight patients.

In our pre-analysis of all patients, splenic volume was not associated with OS in the univariate analysis (**Supplementary Table 1**; **Supplementary Figure 2A**). However, splenic volume was revealed as a significant prognostic factor in multivariate analysis (**Supplementary Table 1**). As the high BMI associated with high SV was strongly associated with favorable prognosis (**Supplementary Figure 2B**), this led us to speculate that the prognostic effect of SV could be masked by BMI. Thus, we divided the total patients into three BMI groups. In underweight, normal-weight and overweight patients, we divided the patients according to median splenic volume and found no significant difference in survival (**Supplementary Figure 3**). Next, we dichotomized the splenic volume into high SV and low SV based on the optimal cutoff of 138 ml, 215 ml and 185 ml, respectively, by identifying the maximum statistics (**Supplementary Figure 4**). High splenic volume was significantly associated with OS, with HR of 3.15 (95% CI, 1.03-9.58) in underweight, 1.55 (95% CI, 1.01-2.39) in normal-weight and 2.80 (95% CI, 1.21-6.47) in overweight patients (**Figures 3A-C**). The prognostic effect of high splenic volume was further validated by multivariate Cox analysis. In the underweight group, however, limited numbers (n=29) precluded us from conducting multivariate analysis for this group. As we further performed analyses in the normal-weight and overweight group, we found that in normal-weight group, high splenic volume was independently associated with a greater risk of death (HR: 1.86, 95% CI: 1.15-3.02, p=0.012) (**Table 2**). In overweight group, high splenic volume also remained an independent factor of OS (HR: 3.55, 95% CI: 1.36-9.26, p=0.010) (**Table 3**). Further, when the patients were divided according to TNM stage, the prognostic effect of high splenic volume was more prominent in stage III-IV patients (**Supplementary Figure 5**).

**Table 2:** Multivariate Cox analysis for OS in normal-weight patients.

Variable		HR (univariable)	HR (multivariable)
Age, y		1.02 (1.01-1.04, p=0.009)	1.03 (1.01-1.05, p=0.001)
Sex	Female	[Reference]	[Reference]
	Male	0.92 (0.61-1.37, p=0.668)	0.70 (0.39-1.28, p=0.250)
BMI, kg/m²		0.90 (0.81-1.01, p=0.082)	0.89 (0.77-1.01, p=0.081)
Tobacco	Never	[Reference]	[Reference]
	Former	1.28 (0.80-2.06, p=0.306)	1.54 (0.82-2.91, p=0.178)
	Current	0.94 (0.57-1.53, p=0.791)	1.31 (0.69-2.46, p=0.408)
Tumor location	Lower third	[Reference]	[Reference]
	Middle thrid	1.58 (1.04-2.41, p=0.034)	1.30 (0.83-2.02, p=0.249)
	Upper third	1.00 (0.54-1.83, p=0.990)	0.98 (0.52-1.86, p=0.962)
Tumor size, cm		1.19 (1.11-1.28, p<0.001)	1.04 (0.94-1.14, p=0.440)
Tumor grade	Poorly differentiated	[Reference]	[Reference]
	Well-moderately differentiated	0.56 (0.35-0.92, p=0.022)	0.72 (0.43-1.22, p=0.226)
TNM Stage	I	[Reference]	[Reference]
	II	2.59 (1.20-5.57, p=0.015)	2.61 (1.20-5.67, p=0.016)
	III	10.96 (5.63-21.35, p<0.001)	9.03 (4.47-18.25, p<0.001)
	IV	23.83 (9.00-63.07, p<0.001)	25.87 (9.19-72.83, p<0.001)
Postoperative complication	No	[Reference]	[Reference]
	Yes	1.35 (0.85-2.13, p=0.200)	1.09 (0.64-1.84, p=0.753)
Splenic volume	< 215 ml	[Reference]	[Reference]
	≥ 215 ml	1.55 (1.01-2.39, p=0.045)	1.86 (1.15-3.02, p=0.012)

**Table 3:** Multivariate Cox analysis for OS in overweight patients.

Variable		HR (univariable)	HR (multivariable)
Age, y		1.05 (1.01-1.09, p=0.026)	1.07 (1.02-1.12, p=0.007)
Sex	Female	[Reference]	[Reference]
	Male	2.32 (0.71-7.63, p=0.164)	1.45 (0.34-6.16, p=0.614)
BMI, kg/m²		0.93 (0.77-1.12, p=0.451)	0.91 (0.73-1.12, p=0.372)
Tobacco	Never	[Reference]	[Reference]
	Former	0.85 (0.30-2.35, p=0.749)	0.88 (0.29-2.65, p=0.817)
	Current	2.00 (0.94-4.27, p=0.072)	2.05 (0.88-4.78, p=0.097)
Tumor location	Lower third	[Reference]	[Reference]
	Middle thrid	3.34 (1.23-9.03, p=0.018)	3.67 (1.11-12.18, p=0.034)
	Upper third	4.77 (1.85-12.33, p=0.001)	5.26 (1.62-17.14, p=0.006)
Tumor size, cm		1.29 (1.10-1.50, p=0.002)	1.12 (0.92-1.36, p=0.262)
Tumor grade	Poorly differentiated	[Reference]	[Reference]
	Well-moderately differentiated	0.56 (0.25-1.25, p=0.157)	0.77 (0.31-1.88, p=0.565)
TNM Stage	I	[Reference]	[Reference]
	II	2.69 (0.90-8.00, p=0.076)	2.20 (0.69-7.05, p=0.185)
	III	6.74 (2.65-17.12, p<0.001)	6.23 (2.09-18.58, p=0.001)
	IV	44.97 (8.75-231.03, p<0.001)	91.89 (11.62-726.88, p<0.001)
Postoperative complication	No	[Reference]	[Reference]
	Yes	1.60 (0.78-3.28, p=0.197)	1.56 (0.67-3.63, p=0.299)
Splenic volume	< 185 ml	[Reference]	[Reference]
	≥ 185 ml	2.80 (1.21-6.47, p=0.016)	3.55 (1.36-9.26, p=0.010)

**Figure 4:**
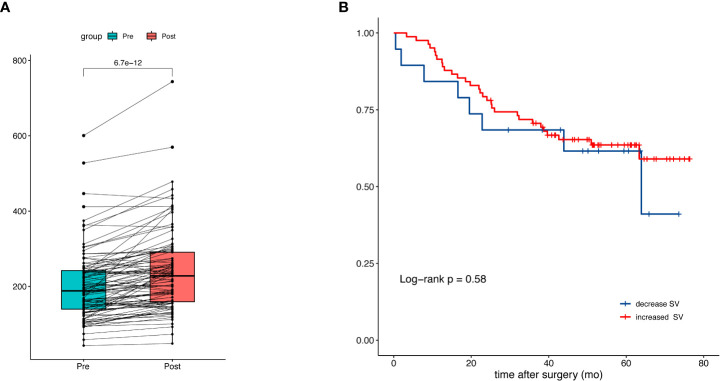
Splenic volume change during neoadjuvant chemotherapy and the prognostic impact. **(A)** Comparison of splenic volume before and after NAC. **(B)** Kaplan-Meier curves of patients with increased and decreased splenic volume.

Next, we investigated the association of △SV during NAC with overall survival. In 101 patients with available CT after NAC, the median (IQR) SV after NAC was 228 (159 to 291) ml versus 188 (140 to 242) ml before NAC (p<0.001) (**Figure 4**). An increase of splenic volume was observed in 82 patients, whereas 19 had decreased splenic volume. The median (range) percentage change of SV was 13.8% (-4.1% to 58.2%). The increase of splenic volume was not significantly associated with OS (HR:0.80; 95% CI: 0.37-1.75) (**Figure 4**).

### Baseline splenic volume and immune status

**Figure 5:**
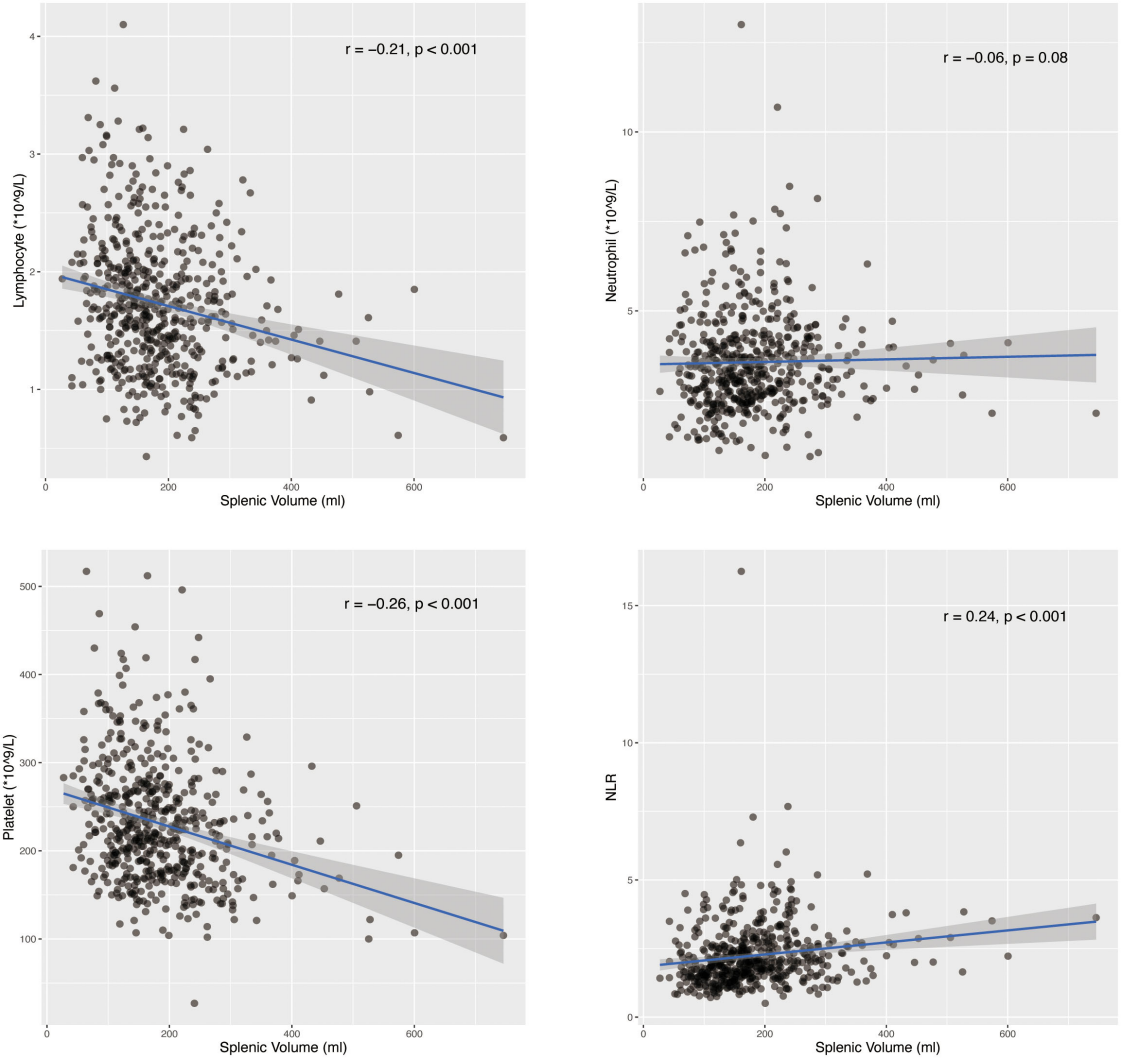
Correlation of splenic volume (ml) and lymphocytes (*10^9/L), neutrophils (*10^9/L), platelets (*10^9/L) and NLR (neutrophil-to-lymphocte ratio).

Platelet and lymphocyte counts were consistently lower in the high SV patients than low SV patients across the three groups. Neutrophil count was generally higher in high SV patients across three groups, but the difference was only significant in normal-weight patients (median [IQR]: 3.7 [2.9-4.6] vs 3.3 [2.6-4.0], p=0.044). We did not find any significant difference in WBC and monocytes. It should be noted that neutrophil to lymphocyte ratio (NLR), which is a measure of systemic inflammation, was greater in patients with high SV (**Supplementary Table 2**). Further, we calculated the Spearman’s correlation in the 541 patients as shown in **Figure 5**. The splenic volume was negatively correlated with lymphocytes (r=-0.21, p<0.001), and positively correlated with NLR (r=0.24, p<0.001). As a reference, the splenic volume was negatively correlated with platelets (r=-0.26, p<0.001). The correlation with neutrophils was slight (r=0.08, p=0.06) (**Figure 5**). Further, we performed analyses in fifty-six patients for lymphocyte subsets. The characteristic of this group of patients was detailed in **Supplementary Table 3**. The SV was negatively correlated with CD4+T cells (r=-0.27, p=0.041) and NK cells (r=-0.30, p=0.025) while the correlation with CD8+T cells and B cells were not significant (**Supplementary Figure 6**).

## Discussion

To our knowledge, this is the first study to investigate the association between baseline splenic volume and the clinical outcome in patients with gastric cancer. The main finding is that patients with low splenic volume had longer overall survival than patients with high splenic volume in a given range of BMI. The negative effect of splenomegaly for overall survival was independent of other prognostic factors, such as age, tumor size and pathological stage. Furthermore, we found a remarkably inverse correlation between baseline splenic volume and the number of circulating lymphocytes. In a subgroup of patients, we found splenic volume tended to inversely correlate with CD4+T cells and NK cells.

Studies have focused on the splenic volume in a few malignancies. In metastatic colorectal cancer, splenic volume > 180 ml was associated with poor PFS (29). In NSCLC, splenic volume > 194 ml was associated with both poor PFS and poor OS (30). Our study supports the prognostic role of splenic volume in gastric cancer. Although splenic volume is proportional to BMI, our finding is unlikely to be biased by the effect of BMI. Gastric cancer patients with higher BMI consistently showed favorable prognosis in our study and other researches (35–38). Gastrectomy can lead to weight loss in patients due to decreased gastric volume and hormone changes. The underlying cause of better prognosis for higher BMI might be related to achieving ideal body weight after gastrectomy and thus better condition in the long term. Excess adipose tissue could also serve as an energy reserve and confers a survival advantage in times of stress and diet restriction. As there is an evident survival advantage with higher BMI, one should have expected a better rather than worse prognosis for patients with high SV instead of the opposite. We found that the cutoff in our study was smaller for underweight patients (138 ml), however, cutoff values reversed for “normal weight” (215 ml) and “overweight” (185 ml) patients. This could be caused by the different composition of the two groups as the overweight group was characterized by less advanced stage patients, and patients with high BMI might have distinct physiology compared with low BMI patients during tumor development. It is possible that these factors could affect the cutoff selection. On the other hand, the median value of splenic volume did not associate with overall survival in our pre-analysis. And despite the positive results with the optimal cutoff values, it is possible that neither the median nor the optimal cutoff could reveal the biologically meaningful classifications. Studies would be warranted to select a more reasonable threshold for splenic volume. It should be acknowledged in our study that the cutoff points should not be translated into risk stratification in the clinical setting, but rather, a reflection of the potential immunosuppressive role of the spleen in gastric cancer patients.

In this study, we also observed a majority of patients underwent spleen enlargement during chemotherapy. The proportion of patients with increased splenic volume during neoadjuvant chemotherapy was similar to that in a previous report of coloretal cancer (39). A known mechanism is chemotherapy-induced hepatic sinusoidal injury, which can result in portal hypertension and the presentation of splenomegaly (39). In spite of this, our study herein showed that the increase in splenic volume does not have a significant impact on prognosis.

It has been found that splenic hematopoietic activity is an important source of tumor-promoting myeloid cells in cancer. In animal models, it was found that the tumor induced an expansion of monocytes with features of myeloid progenitors in a niche of spleen (marginal zone), in which these cells cross-present tumor antigens to memory CD8+T cells and caused immune tolerization (40). It was also discovered that tumor enhanced the capacity of the spleen to recruit granulocyte–macrophage progenitors, which subsequently differentiated into potent myeloid-derived suppressor cells (MDSCs). Splenectomy not only dampened the immunosuppressive function of the myeloid cells but also increased the frequency of infiltrating cytotoxic T cell in tumor microenvironment (41). More recently, it has been identified that a population of erythroid progenitor cells (CD71+TER119+) became dominant in the spleen after tumor establishment, closely resembled MDSCs in transcriptome and impaired effector T cell functions in a similar immunosuppressive way (42). Further, the expansion of CD71+TER119+ erythroid cells in the spleen could be triggered by inflammation-stress and express immune checkpoint molecules, infiltrate tumors, and promote tumor growth (43). Taken together, the spleen can be viewed as a crucial site for extramedullary hematopoiesis and an origin of myeloid lineage with potent immunosuppressive capacities in the context of cancer.

Consistent with the immunosuppressive role of the spleen found in animal studies, we observed an altered peripheral immunity related to the increasing of splenic volume in our study. Neutrophil-to-lymphocyte ratio (NLR) is a negative prognostic indicator in various cancer types (44), as well as a predictor of response to immunotherapy and clinical benefits (45). NLR is also a well defined prognostic index in gastric cancer (46). And a recent study revealed that NLR predicts prognosis in metastatic gastric cancer treated with PD1 inhibitor (47).Neutrophilia is a hallmark of the innate immune response including phagocytosis, release of a variety of cytokines and production of molecular mediators, while lymphocytopenia is a reflection of depressed adaptive immune response, the combination of which measures the intensity of immune-inflammatory response and stress reaction to cancer (48). In our study, we found a more robust association of the splenic volume with lymphocytopenia, indicating a close relationship between the splenic volume and the adaptive arm of the immune system. We also studied the association of splenic volume with two important lymphocyte subsets, CD4+ and CD8+T cells in a small group of patients. While CD8+T cells have powerful killing effects on cancer cells (49), CD4+T cells help CD8+ T cells priming and maturation, so CD4+ T cells must present in the tumor microenvironment for a successful antitumor response (50). It was found that in immunotherapy of gastrointestinal cancer, the decrease of circulating CD4+T cell and CD8+T cell after the first dose of ICIs could indicate poor survival in patients (51). Thus, more emphasis should be put on circulating lymphocyte subsets, especially in the era of immunotherapy. Although we found an inverse correlation between splenic volume and CD4+ T cell counts, this was only observed in a group with limited sample size, and more comprehensive studies that include larger cohorts would be needed to support this association.

As an innate immune cell, NK cell is now regarded as a bridge linking the innate and adaptive immunity as it shapes the adaptive immune response by secreting cytokines (52). A growing number of studies suggested that NK cells can be educated during development, possess antigen-specific receptors, undergo clonal expansion and acquire immunological memory (53). Although high circulating NK cell count was associated with better OS in gastric cancer (23), the prognostic role of NK cells was conflicted in various cancers and warrant further study (54–57).

In conclusion, our study revealed a prognostic role of high splenic volume in gastric cancer and the association of splenic volume with blood immune cells. This is in line with the idea that immune response is coordinated across different tissues, and represents a possible biomarker for immunotherapy benefits.

## Data availability statement

The datasets presented in this article are not readily available because the data contain potentially identifying patient information. Requests to access the datasets should be directed to WK, kangwm@pumch.cn.

## Ethics statement

The studies involving human participants were reviewed and approved by Institutional Review Boards of Peking Union Medical College Hospital. Written informed consent for participation was not required for this study in accordance with the national legislation and the institutional requirements.

## Author contributions

Conceptualization, XY, JY and WK; Data curation, ZZ and ZL; Formal analysis, ZZ, ZL, JL, JS and MM; Writing – original draft, ZZ; Writing – review & editing, ZL, JL, JS, MM, XY, JY and WK.

## Supplementary material

Supplementary Figure 1 Example of splenic volumetry on a CT scan image. A 54-year-old man (BMI: 24.5kg/m2) underwent CT scan for baseline evaluation of gastric cancer. The CT scan shows the wedge-shaped spleen on the axial slice. By manually tracing the boundary of the spleen, the area that was enclosed could be calculated, with the slice thickness **(A)**. The volume of every slice was added up to represent the total volume **(B)**. The splenic volume measured on this CT was 170.87 ml.

Supplementary Figure 2 Kaplan-Meier plot of patients with high and low SV stratified by median value (n=541).

Supplementary Figure 3 Kaplan-Meier curves of patients stratified by median splenic volume in **(A)** underweight **(B)** normal-weight and **(C)** overweight group.

Supplementary Figure 4 Selection of the optimal cutoff point using maxstat R package. In underweight **(A)**, normal-weight **(B)** and overweight **(C)** group, patients were divided into two groups based on a candidate splenic volume cutoff. The X-axis indicates the candidate cutoff point of splenic volume (ml). The Y-axis reports the corresponding standardized log-rank statistic values. Different splenic volume were examined as candidate cutoff points and the value that best separates survival curves was selected as the optimal cutoff. The vertical dotted line indicated the splenic volume that generated the maximum standardized log-rank statistics and minimum p value. As is shown in the plots, the optimal cutoffs for underweight, normal-weight and overweight group were 138 ml **(A)**, 215 ml **(B)** and 185 ml **(C)**, respectively.

Supplementary Figure 5 Kaplan-Meier curves of patients with high and low SV stratified by TNM stage in **(A)** underweight **(B)** normal-weight and **(C)** overweight group.

Supplementary Figure 6 Correlation of splenic volume and lymphocyte subsets.
