# Supplementary material for: Baseline splenic volume as a biomarker for clinical outcome and circulating lymphocyte count in gastric cancer
Source: Front Oncol. 2023 Jan 30;12:1065716. doi: 10.3389/fonc.2022.1065716 (PMC9923954; doi:10.3389/fonc.2022.1065716)
Supplement: Supplementary file 1 [file DataSheet_1.docx]

**Supplementary Table 1.** Multivariate Cox analysis in all patients (n=541)

| Variables |  | HR (univariable) | HR (multivariable) |
| --- | --- | --- | --- |
| Age |  | 1.02 (1.009-1.041, p=0.003) | 1.03 (1.014-1.047, p<0.001) |
| Sex | Female | [Reference] | [Reference] |
|  | Male | 0.99 (0.691-1.407, p=0.938) | 0.77 (0.475-1.244, p=0.284) |
| BMI, kg/m² |  | 0.89 (0.848-0.941, p<0.001) | 0.89 (0.837-0.940, p<0.001) |
| Tobacco | Never | [Reference] | [Reference] |
|  | Former | 1.27 (0.842-1.905, p=0.257) | 1.57 (0.955-2.594, p=0.075) |
|  | Current | 1.29 (0.876-1.887, p=0.199) | 1.43 (0.904-2.273, p=0.126) |
| Tumor location | Lower third | [Reference] | [Reference] |
|  | Middle third | 1.64 (1.134-2.371, p=0.009) | 1.50 (1.026-2.204, p=0.036) |
|  | Upper third | 1.42 (0.928-2.166, p=0.106) | 1.56 (1.002-2.413, p=0.049) |
| Tumor size, cm |  | 1.22 (1.149-1.306, p<0.001) | 1.06 (0.985-1.151, p=0.116) |
| Tumor grade | Poorly differentiated | [Reference] | [Reference] |
|  | Well-moderately differentiated | 0.55 (0.370-0.818, p=0.003) | 0.72 (0.474-1.107, p=0.136) |
| TNM Stage | I | [Reference] | [Reference] |
|  | II | 3.03 (1.649-5.550, p<0.001) | 2.62 (1.413-4.852, p=0.002) |
|  | III | 10.59 (6.218-18.039, p<0.001) | 7.76 (4.411-13.637, p<0.001) |
|  | IV | 31.76 (14.293-70.565, p<0.001) | 31.82 (13.827-73.211, p<0.001) |
| Postoperative complications | No | [Reference] | [Reference] |
|  | Yes | 1.28 (0.884-1.850, p=0.192) | 1.25 (0.842-1.859, p=0.267) |
| Splenic volume, ml |  | 1.00 (0.999-1.003, p=0.363) | 1.00 (1.001-1.005, p=0.005) |

**Supplementary Table 2.** Comparison of immune related parameters in patients with high and low splenic volume

|  | underweight | |  | Normal-weight | |  | Overweight | |  |
| --- | --- | --- | --- | --- | --- | --- | --- | --- | --- |
|  | low splenic volume | high splenic volume | p | low splenic volume | high splenic volume | p | low splenic volume | high splenic volume | p |
| White blood cell, 10^9/L | 5.2 (4.5-6.2) | 5.3 (4.6-6.3) | 0.63 | 5.6 (4.7-6.6) | 5.6 (4.8-7.0) | 0.5 | 6.0 (5.2-7.0) | 6.0 (4.9-6.9) | 0.66 |
| Platelet, 10^9/L | 242.0 (220.5-280.5) | 209.5 (154.0-252.5) | 0.06 | 230.5 (190.5-273.8) | 192.0 (160.5235.2) | <0.001 | 226.0 (197.0-279.0) | 212.0 (170.0-245.0) | 0.005 |
| Monocyte, 10^9/L | 0.3 (0.2-0.4) | 0.3 (0.3-0.3) | 0.3 | 0.3 (0.3-0.4) | 0.3 (0.2-0.4) | 0.36 | 0.4 (0.3-0.4) | 0.3 (0.3-0.4) | 0.13 |
| Neutrophil, 10^9/L | 2.8 (2.4-3.6) | 3.5 (2.8-4.2) | 0.14 | 3.3 (2.6-4.0) | 3.7 (2.9-4.6) | 0.044 | 3.5 (2.8-4.2) | 3.7 (2.7-4.3) | 0.64 |
| Lymphocyte, 10^9/L | 1.9 (1.2-2.1) | 1.4 (1.0-1.5) | 0.06 | 1.7 (1.4-2.0) | 1.5 (1.1-2.1) | 0.037 | 1.8 (1.4-2.4) | 1.7 (1.3-1.9) | 0.036 |
| NLR | 1.5 (1.2-2.1) | 2.9 (1.9-3.4) | 0.025 | 1.9 (1.5-2.5) | 2.4 (1.8-3.2) | 0.001 | 1.9 (1.5-2.6) | 2.1 (1.7-2.8) | 0.09 |

**Supplementary Table 3.** Patient characteristics of subgroup with lymphocyte subsets (n=56)

| Age, median (IQR), y | 56.5 (49.5 to 64.2) |
| --- | --- |
| Male | 40 (71.4) |
| BMI, median (IQR), kg/m² | 22.8 (20.6 to 24.9) |
| BMI group |  |
| Underweight | 4 (7.1) |
| Normal-weight | 38 (67.9) |
| Overweight | 14 (25.0) |
| Tobacco |  |
| Never | 29 (51.8) |
| Former | 18 (32.1) |
| Current | 9 (16.1) |
| Neoadjuvant chemotherapy | 42 (75.0) |
| Tumor location |  |
| Lower third | 35 (62.5) |
| Middle thrid | 14 (25.0) |
| Upper third | 7 (12.5) |
| Tumor size, median (IQR), cm | 2.4 (1.8 to 4.0) |
| Stage |  |
| I | 19 (33.9) |
| II | 19 (33.9) |
| III | 16 (28.6) |
| IV | 2 (3.6) |
| Adjuvant chemotherapy | 43 (76.8) |
| Baseline splenic volume, median (IQR), ml | 180.4 (99.7) |
