# Supplementary figures and images for: Baseline splenic volume as a biomarker for clinical outcome and circulating lymphocyte count in gastric cancer
Source: Front Oncol. 2023 Jan 30;12:1065716. doi: 10.3389/fonc.2022.1065716 (PMC9923954; doi:10.3389/fonc.2022.1065716)

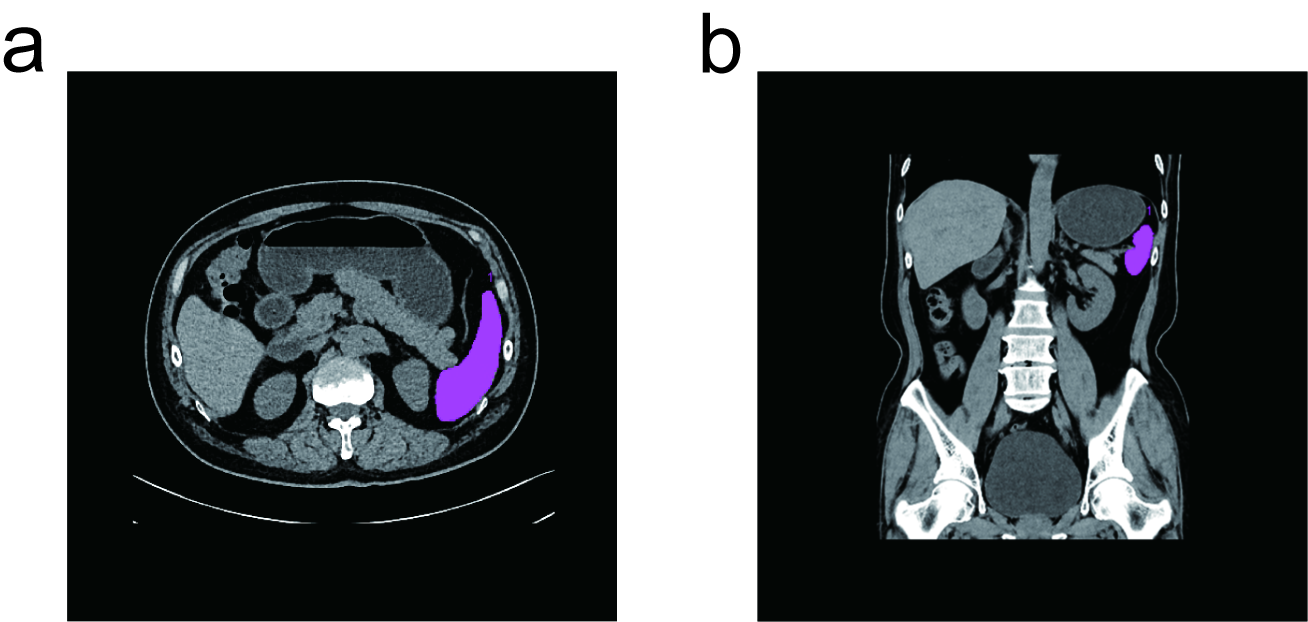

Supplement: Supplementary Figure 1 — Example of splenic volumetry on a CT scan image. A 54-year-old man (BMI: 24.5kg/m2) underwent CT scan for baseline evaluation of gastric cancer. The CT scan shows the wedge-shaped spleen on the axial slice. By manually tracing the boundary of the spleen, the area that was enclosed could be calculated, with the slice thickness (A). The volume of every slice was added up to represent the total volume (B). The splenic volume measured on this CT was 170.87 ml. [file Image_1.tif]

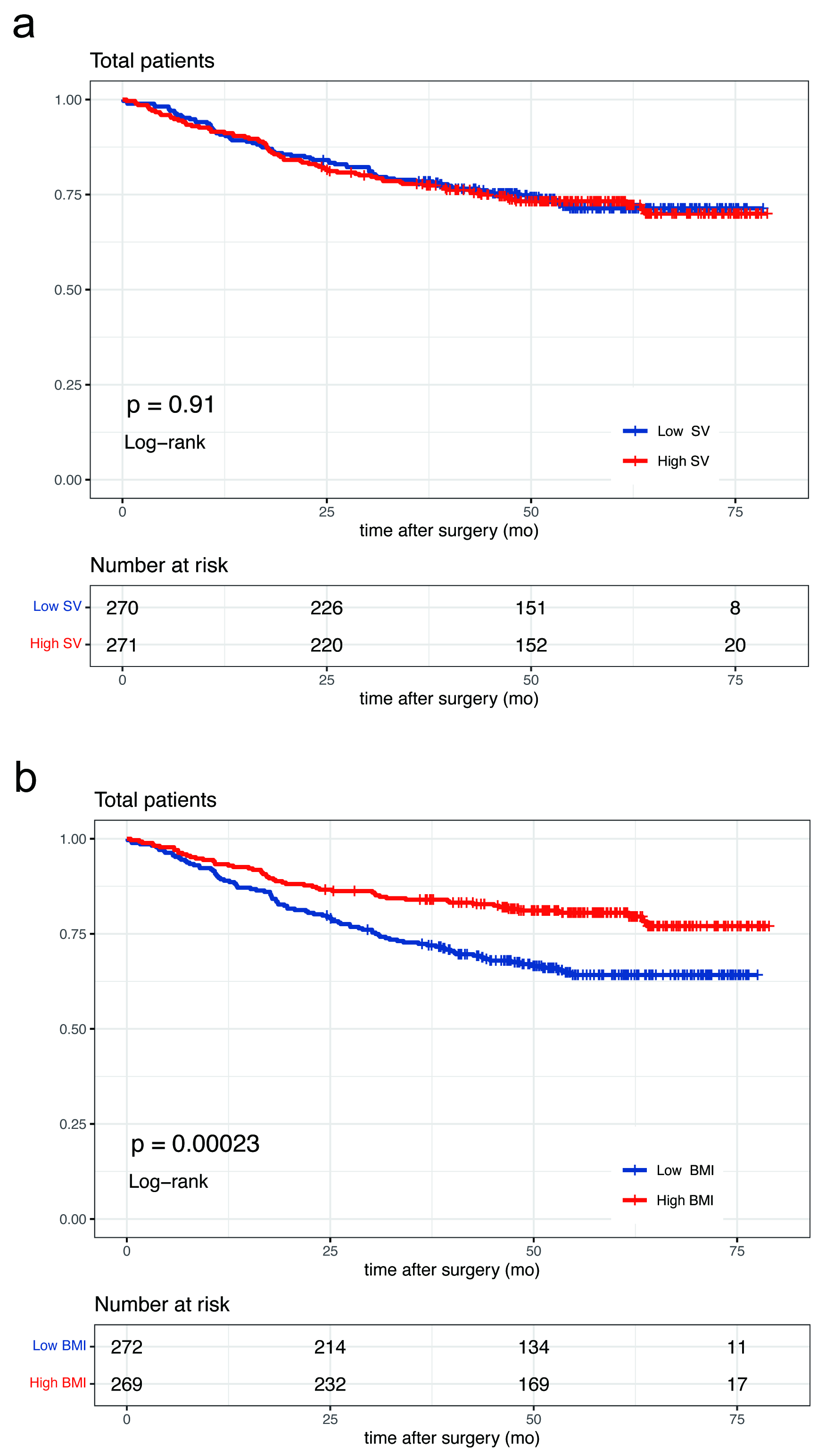

Supplement: Supplementary Figure 2 — Kaplan-Meier plot of patients with high and low SV stratified by median value (n=541). [file Image_2.tif]

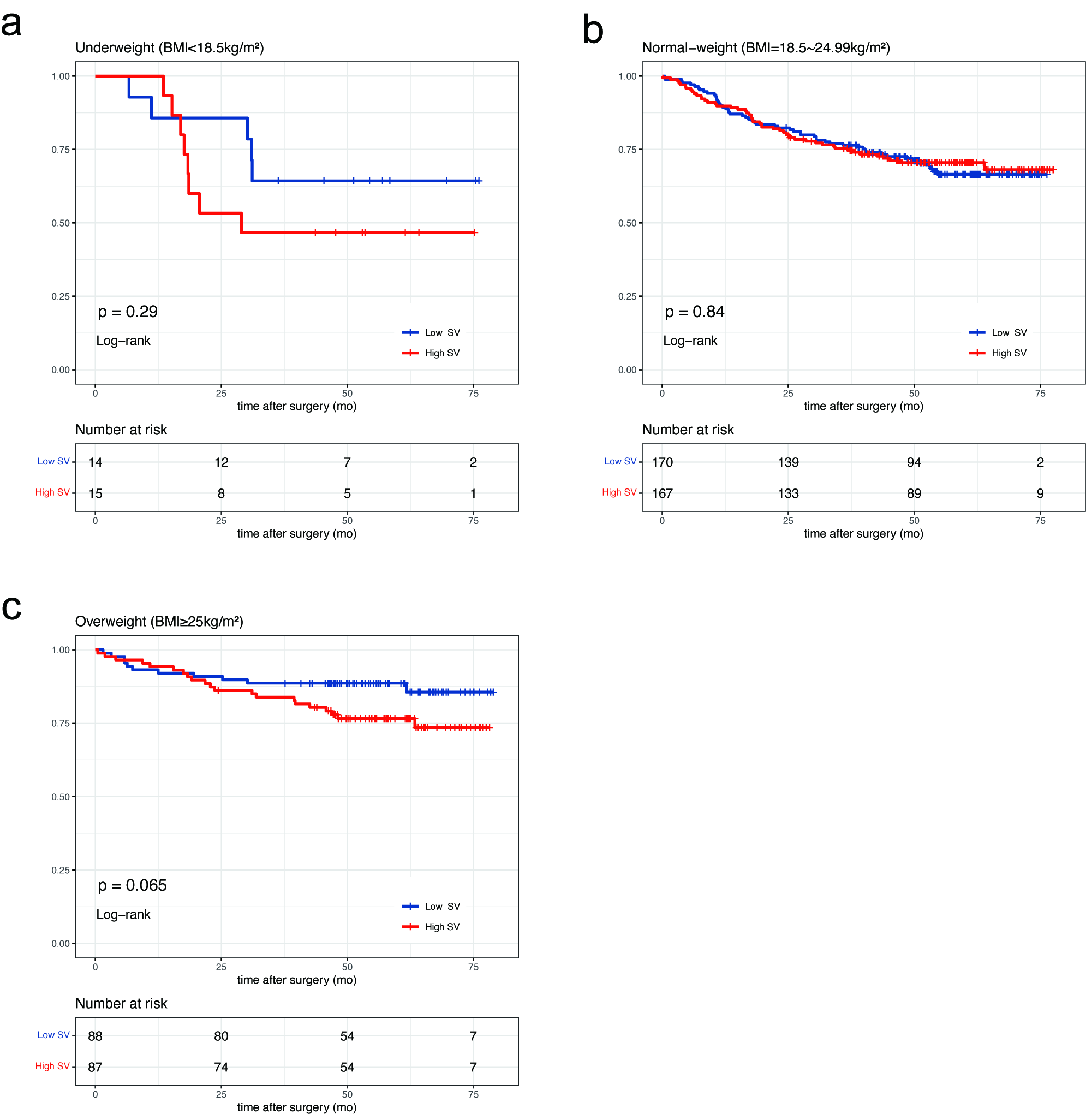

Supplement: Supplementary Figure 3 — Kaplan-Meier curves of patients stratified by median splenic volume in (A) underweight (B) normal-weight and (C) overweight group. [file Image_3.tif]

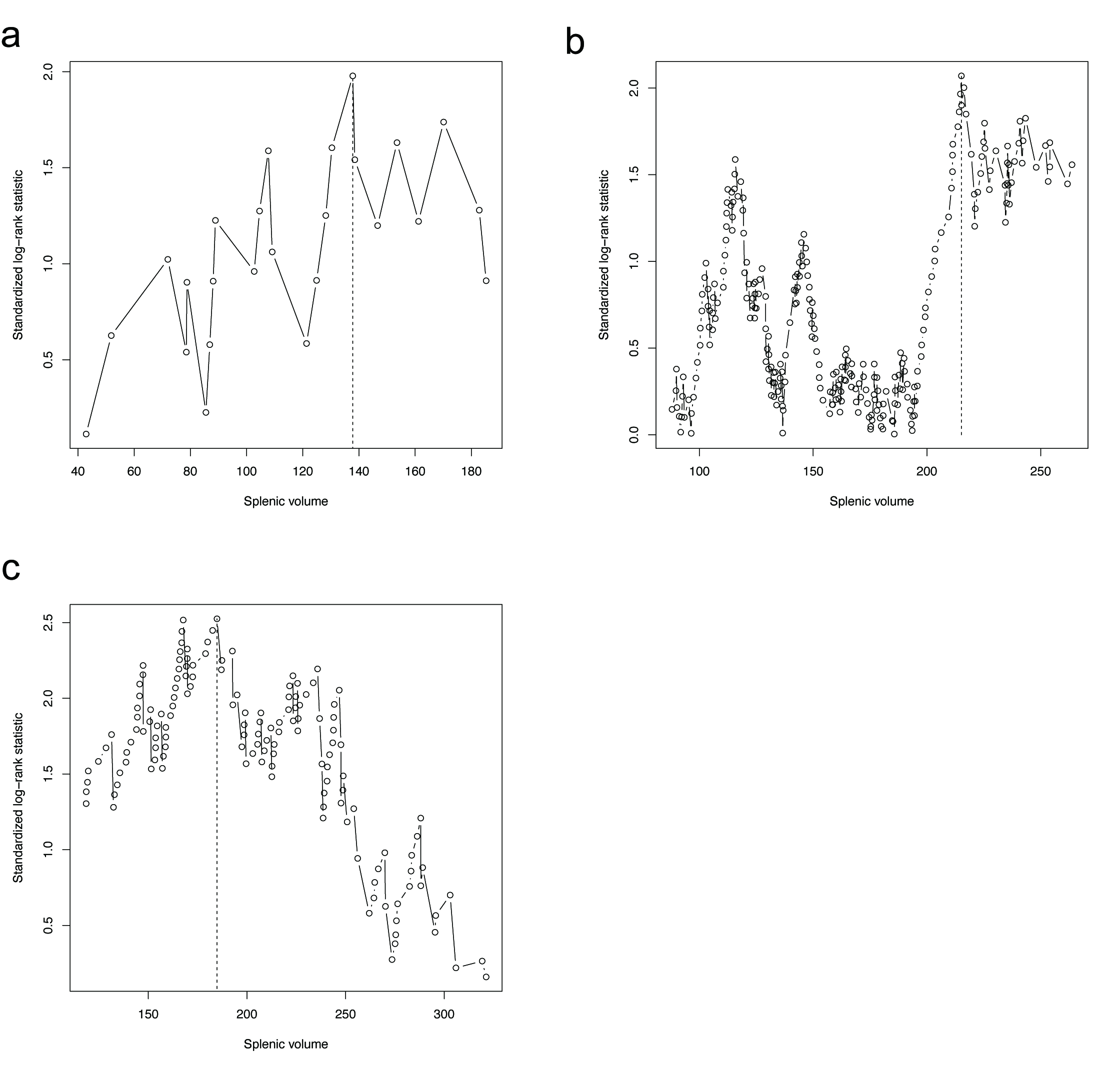

Supplement: Supplementary Figure 4 — Selection of the optimal cutoff point using maxstat R package. In underweight (A), normal-weight (B) and overweight (C) group, patients were divided into two groups based on a candidate splenic volume cutoff. The X-axis indicates the candidate cutoff point of splenic volume (ml). The Y-axis reports the corresponding standardized log-rank statistic values. Different splenic volume were examined as candidate cutoff points and the value that best separates survival curves was selected as the optimal cutoff. The vertical dotted line indicated the splenic volume that generated the maximum standardized log-rank statistics and minimum p value. As is shown in the plots, the optimal cutoffs for underweight, normal-weight and overweight group were 138 ml (A), 215 ml (B) and 185 ml (C), respectively. [file Image_4.tif]

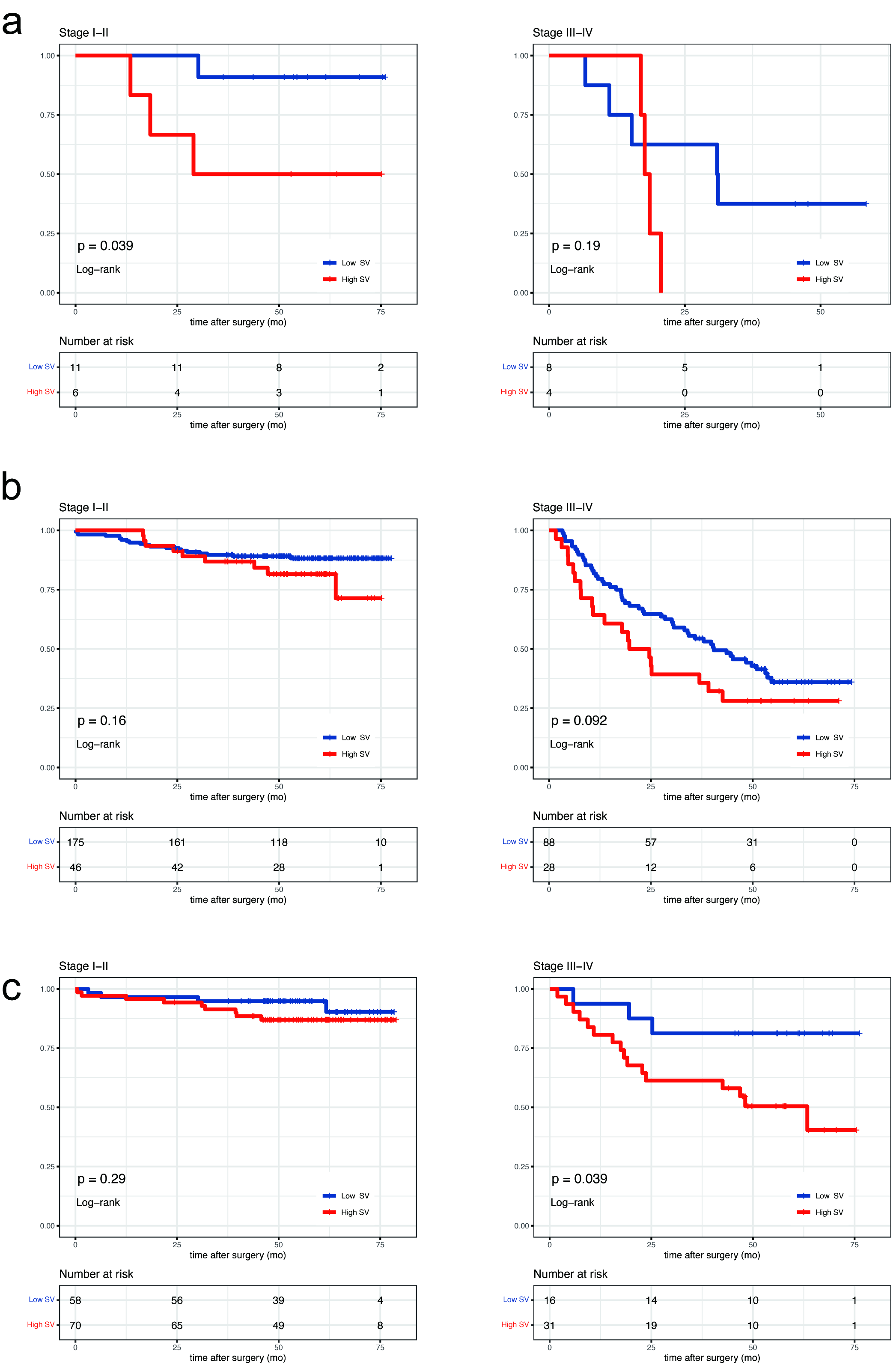

Supplement: Supplementary Figure 5 — Kaplan-Meier curves of patients with high and low SV stratified by TNM stage in (A) underweight (B) normal-weight and (C) overweight group. [file Image_5.tif]

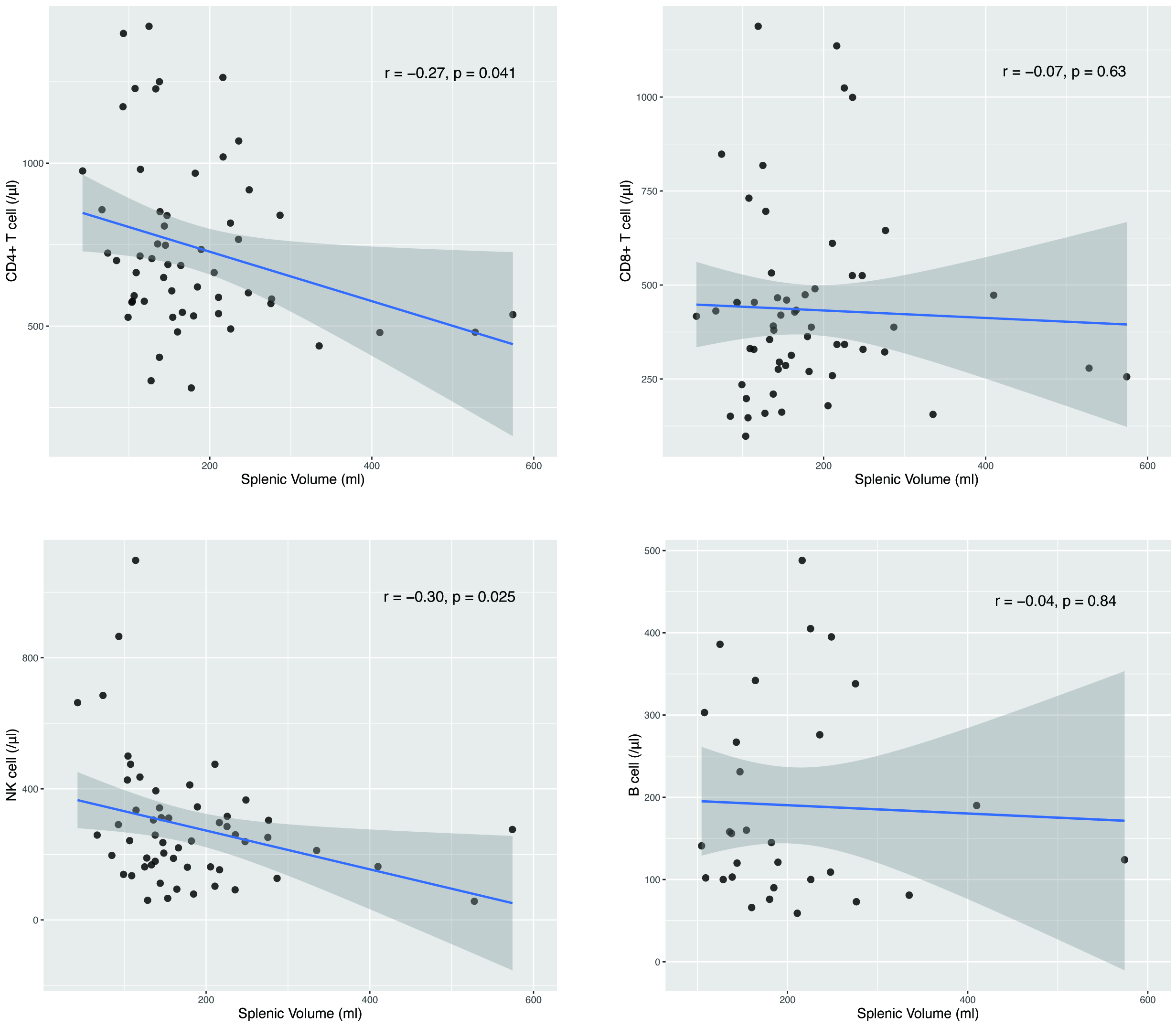

Supplement: Supplementary Figure 6 — Correlation of splenic volume and lymphocyte subsets. [file Image_6.tif]
